# *Eimeria tenella* apical membrane 2 overexpression enhances pathogenicity and immunity in chickens

**DOI:** 10.1186/s13071-025-07185-0

**Published:** 2025-12-06

**Authors:** Shanbo Wu, Lihui Wang, Jinwen Wang, Wenqi Han, Jiayu Bai, Fanghe Zhao, Yu Yu, Qiping Zhao, Shunhai Zhu, Hongyu Han, Hui Dong

**Affiliations:** Key Laboratory of Animal Parasitology of Ministry of Agriculture, Shanghai Veterinary Research Institute, Chinese Academy of Agricultural Sciences, Minhang, Shanghai 200241 People’s Republic of China

**Keywords:** *Eimeria tenella*, *Et*AMA2, Overexpression, Virulence, Immunogenicity

## Abstract

**Background:**

*Eimeria tenella*, a highly pathogenic apicomplexan parasite, causes severe avian coccidiosis, threatening global poultry production. Apical membrane antigens (AMAs), conserved proteins in apicomplexan parasites, are critical for host cell invasion, making them promising vaccine targets. In this study, we constructed an the overexpression strain of *E. tenella* AMA2 (*Et*AMA2-OE) and evaluated its pathogenicity and immunogenicity.

**Methods:**

Transcriptional profiling of *Et*AMA2 during infection was conducted using reverse transcription quantitative real-time polymerase chain reaction (RT-qPCR). A homozygous *Et*AMA2-OE strain was generated using plasmid transfection, drug selection, and flow cytometry. Pathogenicity was assessed through in vitro sporozoite invasion assays and in vivo evaluations, including cecal lesion scoring, oocyst shedding, and weigh-gain monitoring in chicks. Furthermore, immunogenicity was evaluated by challenging immunized chicks with wild-type *E. tenella*.

**Results:**

The *Et*AMA2-OE strain significantly enhanced invasion efficiency and pathogenicity, causing severe cecal pathology, increased oocyst output, and weight loss. Importantly, immunization with *Et*AMA2-OE conferred substantial immunity, resulting in significantly reduced oocyst shedding and markedly attenuated cecal lesions in immunized chicks.

**Conclusions:**

The present data indicated that *Et*AMA2 had dual functions as a virulence factor critical for early invasion and a promising vaccine antigen. This study thereby provided new insights for future drug target screening and development of a vaccine against coccidiosis.

**Graphical Abstract:**

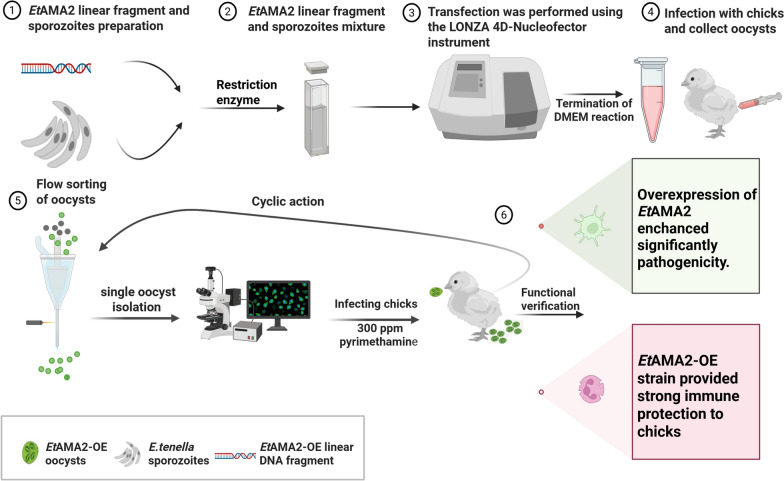

**Supplementary Information:**

The online version contains supplementary material available at 10.1186/s13071-025-07185-0.

## Background

Chicken coccidiosis is an intestinal disease caused by *Eimeria* species, with *Eimeria tenella* being the most pathogenic among the seven prevalent [1, 2]. After invading the host’s intestinal epithelial cells, the sporozoites develop into schizonts and undergo multiple rounds of schizogony, causing significant damage to the host, including hemorrhagic disease and death [3]. Vaccination is a widely recognized preventive strategy against these infections. For example, live non-attenuated vaccines, such as CocciVac and Immucox, are widely used and have been reported to be effective against coccidiosis [4, 5]. However, critical challenges persist regarding the potential risks of live non-attenuated vaccine-induced disease and the production costs of vaccines [6–8]. The use of precocious attenuated *Eimeria* strains as live attenuated vaccines to immunize chickens has a good protective effect, but it also causes slight damage to the intestinal mucosa and delays the weight gain of infected chickens [9, 10]. Therefore, it is critically important to screen protective antigens to develop new subunit vaccines targeting coccidiosis.

The characterization of *Eimeria*-derived immunogens, notably apical membrane antigen 1 (AMA1) and immune-mapped Protein 1 (IMP1), has revolutionized anticoccidial strategies by enabling the development of recombinant subunit vaccines with targeted efficacies in poultry [11, 12]. The identification of suitable antigenic targets for incorporation into subunit vaccines poses a significant challenge, particularly for complex pathogens with intricate antigenic profiles [13, 14]. Consequently, the systematic investigation of the immunological functions of candidate antigens in *Eimeria* species is crucial for the development of effective anticoccidial vaccines [15, 16].

The apical membrane antigens (AMAs) form a class of highly conserved microneme proteins that play key roles in the invasion of host cells and have been proposed as potential vaccine targets [17–21]. Previous studies in apicomplexa protozoa suggest that AMAs exhibit functional specialization during distinct developmental phases. Among them, AMA1 has been extensively characterized in *Plasmodium* and *Toxoplasma gondii*, in which it interacts with rhoptry neck protein (RON2) to drive invasion and modulate host immune responses [22, 23]. For example, *Plasmodium* AMA1 is indispensable for merozoite invasion of erythrocytes, whereas *T. gondii* AMA1 is critical for tachyzoite motility and host cell penetration [24, 25]. In *E. tenella*, four AMA paralogs (*Et*AMA1–4) have been identified. *Et*AMA1 is a sporozoite-specific protein involved in the invasion of sporozoites, and the delivery of the *Et*AMA1 antigen by *Lactococcus lactis* induces robust immune responses against *E. tenella* infection [19, 26, 27]. Despite these advances, no systematic investigation has addressed whether *E. tenella*’s other AMA paralogs similarly exhibit stage-specific functions and immunogenicity. *Et*AMA2, which shows peak transcription during the early stage of infection, may mainly be involved in the invasion and schizogony stage [28, 29]. To verify whether *Et*AMA2 had an immune function suitable for a candidate antigen, we generated a homozygous *Et*AMA2-overexpression strain, *Et*AMA2-OE, and evaluated its invasion efficiency, pathogenicity, and immunogenicity. This study advanced our understanding of AMA family diversification in apicomplexa protozoa and also provided a theoretical basis for the screening of vaccine antigens for avian coccidiosis.

## Methods

### Parasites and animals

The *E. tenella* strain (Resource Number CAAS21111601) was maintained in our laboratory, passaged through chicks every 3–4 months, and stored in 2.5% potassium dichromate at 4 °C after purification. One-day-old Yellow Feather chicks were purchased from the Shanghai Minyou Poultry Breeding Cooperative, raised in isolators equipped with high-efficiency particulate air (HEPA) filters, and provided with coccidia-free feed and water.

All animal procedures were approved by the Animal Ethics Committee of the Shanghai Veterinary Institute, Chinese Academy of Agricultural Sciences, adhering to ethical guidelines and approved protocols (Permit Number: SHVRI-SZ-20230323-4).

### Cell culture model and *E. tenella* infection

Chicken embryo fibroblast cells (DF-1) were cultured in Dulbecco’s modified Eagle’s medium supplemented with 10% fetal bovine serum, 4 mmol/L L-glutamine, 100 U/mL penicillin, and 100 U/mL streptomycin at 37 °C in a 5% CO_2_ atmosphere. This cell line served primarily as an in vitro model for *E. tenella* infection.

The purified unsporulated oocysts were sporulated at 28 °C in 2.5% potassium dichromate. Sporozoites were then harvested from sporulated oocysts following in vitro excystation with bile-trypsin treatment and Percoll gradient centrifugation. These sporozoites were subsequently added to a cell monolayer in 24-well plates at a sporozoite-to-cell ratio of 3:1.

### RT-qPCR

The expression profile of *Et*AMA2 (ETH2_0816100) messenger RNA (mRNA) was examined at various time points during *E. tenella* infection using reverse transcription quantitative real-time polymerase chain reaction (RT-qPCR). Seven-day-old chicks were inoculated with 2.0 × 10^4^ sporulated oocysts of *E. tenella*. At each time point, the entire cecum was collected from three birds, rinsed with phosphate-buffered saline (PBS) to remove luminal contents, and subsequently used for total RNA extraction with TRIzol Reagent (Invitrogen, Waltham, MA, USA) in accordance with the manufacturer’s instructions, and then treated with recombinant DNase I (Takara, Kyoto, Japan). The housekeeping 18S ribosomal RNA (rRNA) of the *E. tenella* gene was used as the internal control. Gene expression was quantified using SYBR^®^ Green Real-time PCR Master Mix (Vazyme Biotech, China). Differences (fold changes) in gene expression were calculated using the 2^−ΔΔCt^ method to determine the relative expression of each RNA. The primer sequences used in this study are listed in Supplementary Table S7. All the experiments were conducted independently in triplicate.

### Construction of the *Et*AMA2-overexpression strain

The *Et*AMA2 gene (1614 bp) was amplified from the first strand of sporulated oocyst complementary DNA (cDNA) using specific primers with homologous arms. The target gene was then ligated to the overexpression vector stored in our laboratory to create the recombinant *Et*AMA2-OE plasmid.

The *Et*AMA2-OE plasmid was linearized, and 25 μg of the purified target fragment was combined with 5 × 10^6^ sporozoites in electroporation buffer. Transfection was performed using the LONZA 4D-Nucleofector instrument (LONZA, Germany). The transfected sporozoites were then introduced into 7-day-old chicks via the cloaca. At 12 hpi, chicks were provided with water and feed supplemented with 150 ppm pyrimethamine. Fresh fecal specimens containing unsporulated oocysts were collected 7–8 days postinfection. The oocysts were then purified and allowed to sporulate as previously described [30]. After sporulation, EYFP-positive parasites were sorted by flow cytometry and orally inoculated into 7-day-old coccida-free chicks, which were then fed water and feed containing 300 ppm pyrimethamine. This process was repeated four times, and then the chicks were infected with an oocyst using the single oocyst isolation technique, which ultimately yielded a homozygous *Et*AMA2-OE strain [31]. Then, the *Et*AMA2-OE strain underwent several rounds of in vivo propagation in chicks. A Western blot analysis was performed to detect *Et*AMA2 protein expression using a Flag-labeled antibody. All the primer sequences and plasmid profiles used in this study are provided in Supplementary Table S8 and Supplementary Fig. S1.

### Western blotting

The total proteins from sporozoites of the *Et*AMA2-OE strain were extracted using RIPA Lysis Buffer (Solarbio Life Sciences, Beijing, China). The protein concentrations were measured using a Pierce™ BCA Protein Assay Kit (Thermo Fisher Scientific, Waltham, MA, USA). In total, 15 μg of protein was separated using 10% sodium dodecyl sulfate–polyacrylamide gel electrophoresis (SDS-PAGE) and then transferred to polyvinyl difluoride membranes (0.45 μm) (Millipore, Billerica, MA, USA). The membranes were blocked with 5% skimmed milk (in PBS containing Tween 20 [PBST]) for 2 h and then incubated overnight with the primary antibody at 4 °C. The antibodies used included rabbit antibodies directed against anti-Flag antibodies (1:10,000; Sigma), or α-tubulin (1:5000; Sigma), and the membranes were then incubated with a horseradish-peroxidase-conjugated secondary goat anti-rabbit antibody (diluted 1:5,000; Immunoway) at room temperature for 1–2 h. The protein bands were visualized using an enhanced chemiluminescence system.

### The invasion efficiencies of sporozoites

Sporozoites of the maternal *E. tenella* Shanghai strain (*Et*-Sh) were fluorescently labeled following the instructions of the Vybrant^®^ carboxyfluorescein diacetate succinimidyl este (CFDA-SE) cell tracer kit (Thermo Fisher Scientific Inc, America). Due to the intrinsic EYFP^+^ green fluorescence of the *Et*AMA2-OE strain, *Et*AMA2-OE sporozoites were not labeled; however, the same treatment steps were conducted using PBS as a control. A monolayer of 80–90% confluent DF-1 cells in 24-well plates was then infected with sporozoites at a sporozoite-to-cell ratio of 3:1 for 8 h. At 8 hpi, cells were washed with PBS, digested with trypsin for 5 min, and centrifuged at 800 × *g* for 5 min at room temperature. The supernatant was discarded, and the pellet was washed twice with PBS. The pellet was then resuspended in 50 μL PBS and analyzed. Sporozoite invasion rates were subsequently measured using flow cytometry. All the experiments were conducted independently in triplicate.

### Evaluation of *Et*AMA2-OE pathogenicity

Briefly, 14-day-old chicks having similar body weights (BW) were divided into three groups, with eight chicks per group. Two groups were orally inoculated with equal amounts of sporulated oocysts of either the *Et*-Sh strain or the *Et*AMA2-OE strain (2.0 × 10^4^ oocysts per chick). The third group contained the healthy controls. The mean weight gain (WG) during infection, oocyst production, cecal lesion scores, and mortality rates were used as evaluation indices. WG = final BW − initial BW. Fecal samples were weighed after removing the tray weight. Samples were uniformly collected from the four corners and the center of the fecal tray. For each group, feces were thoroughly mixed, and 2 g was transferred to a beaker. Then, 58 mL of water was added (30-fold dilution per gram of feces). The mixture was filtered through gauze, and 2 mL of the filtrate were mixed with 4 mL of saturated saline solution (resulting in an additional 3-fold dilution, i.e., 90-fold dilution in total). Total oocyst production = total of daily oocyst shedding per bird from 6 to 8 dpi; daily oocyst shedding per bird = oocysts per gram of stool (OPG) × fecal weight/number of bird [27]. Cecal lesions were scored in accordance with the method of Johnson and Reid (1970). Chickens that died from coccidiosis during the experiment were assigned a cecal lesion score of 4. The virulence of the *Et*AMA2-OE strain was evaluated by comparison with the *Et*-Sh strain in terms of oocyst production, lesion severity, and other changes.

### Assessment of the immunogenicity of *Et*AMA2-OE

Briefly, 7-day-old chicks having similar body weights were divided into four groups—*Et*AMA2-OE, *Et*-Sh, nonimmune infection, and healthy control, with eight chicks per group. The *Et*AMA2-OE and *Et*-Sh groups were orally inoculated with the same amount of sporulated oocysts (0.1 × 10^4^ oocysts per chick) from the *Et*AMA2-OE and *Et*-Sh strains, respectively. At 14 days post-immunization, all the groups, except the healthy controls, were challenged with *Et*-Sh sporulated oocysts (2.0 × 10^4^ oocysts per chick). The immune protection conferred by the *Et*AMA2-OE strain was evaluated on the basis of mean weight gain, oocyst production, cecal lesion score, and mortality following the challenge.

### Statistics and reproducibility

All statistical analyses were performed with GraphPad Prism 8.0.2 (GraphPad Software, San Diego, CA, USA). Differences between two groups were analyzed with an unpaired *t*-test with Bonferroni’s correction, and differences among multiple groups were analyzed with nonparametric one-way analysis of variance (ANOVA). All data are expressed as means ± standard deviations (SD). All experiments were performed with at least three biological replicates. For all analyses, *P* < 0.05 was considered significant. (*P < 0.05, **P < 0.01, and ***P < 0.001, ^a,b,c,d^*P* < 0.05.)

## Results

### The *Et*AMA2 transcription level at different developmental stages

**Fig. 1:**
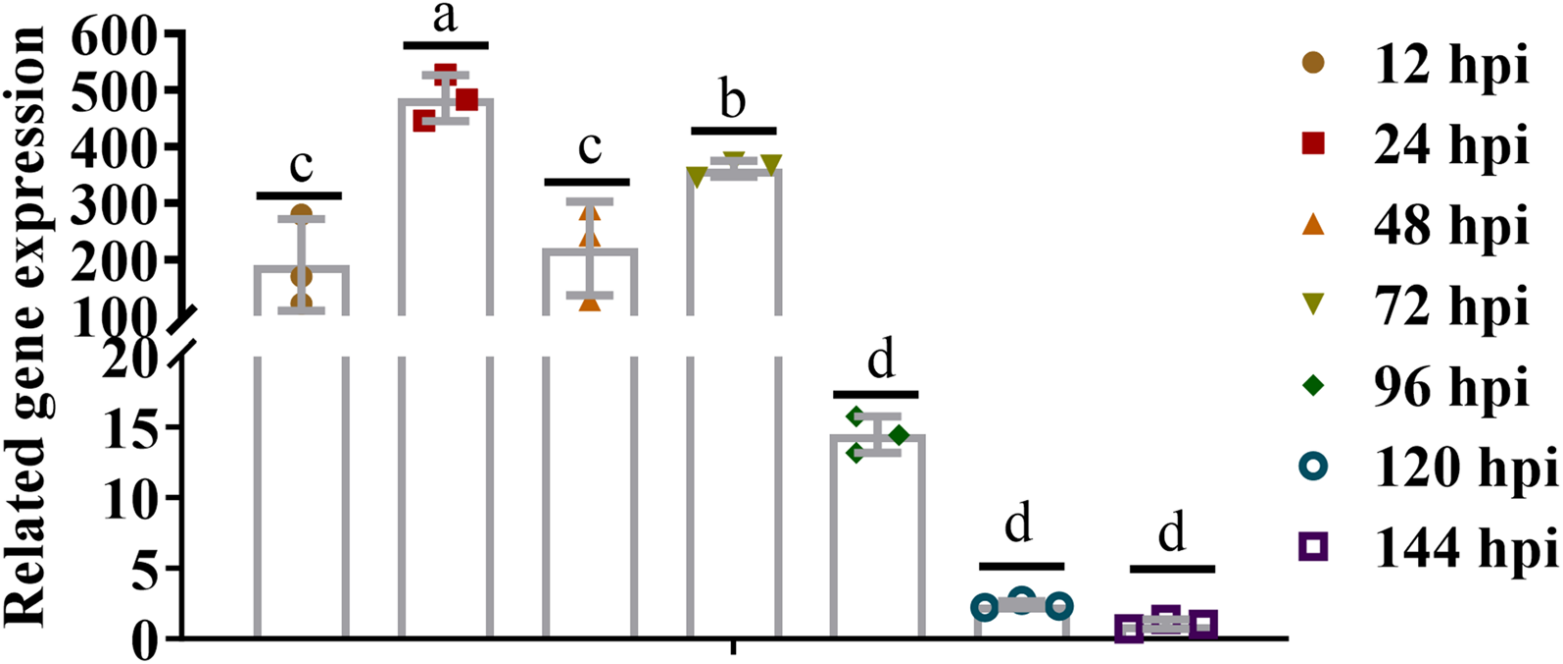
The mRNA transcription levels of *Et*AMA2 at different developmental stages. Transcription levels of *Et*AMA2 in chicken cecum at different times after *E. tenella* infection, determined with RT-qPCR (*n* = 3 biologically independent samples, ^a,b,c,d^*P* < 0.05)

RT-qPCR was performed to measure the mRNA transcriptional levels of *Et*AMA2 in chicken cecum at 0, 12, 24, 48, 72, 96, 122, and 144 hpi. *Et*AMA2 exhibited a high mRNA transcriptional level during the early infection phase (12–72 h), peaking at 24 hpi, whereas during the late stage of infection (96–144 h), the transcriptional level was low (Fig. 1). This revealed that the *Et*AMA2 transcription levels during the parasitic developmental stages were dynamic and mainly involved in the early *E. tenella* developmental stages.

### Construction and identification of the *Et*AMA2-overexpression strain

**Fig. 2:**
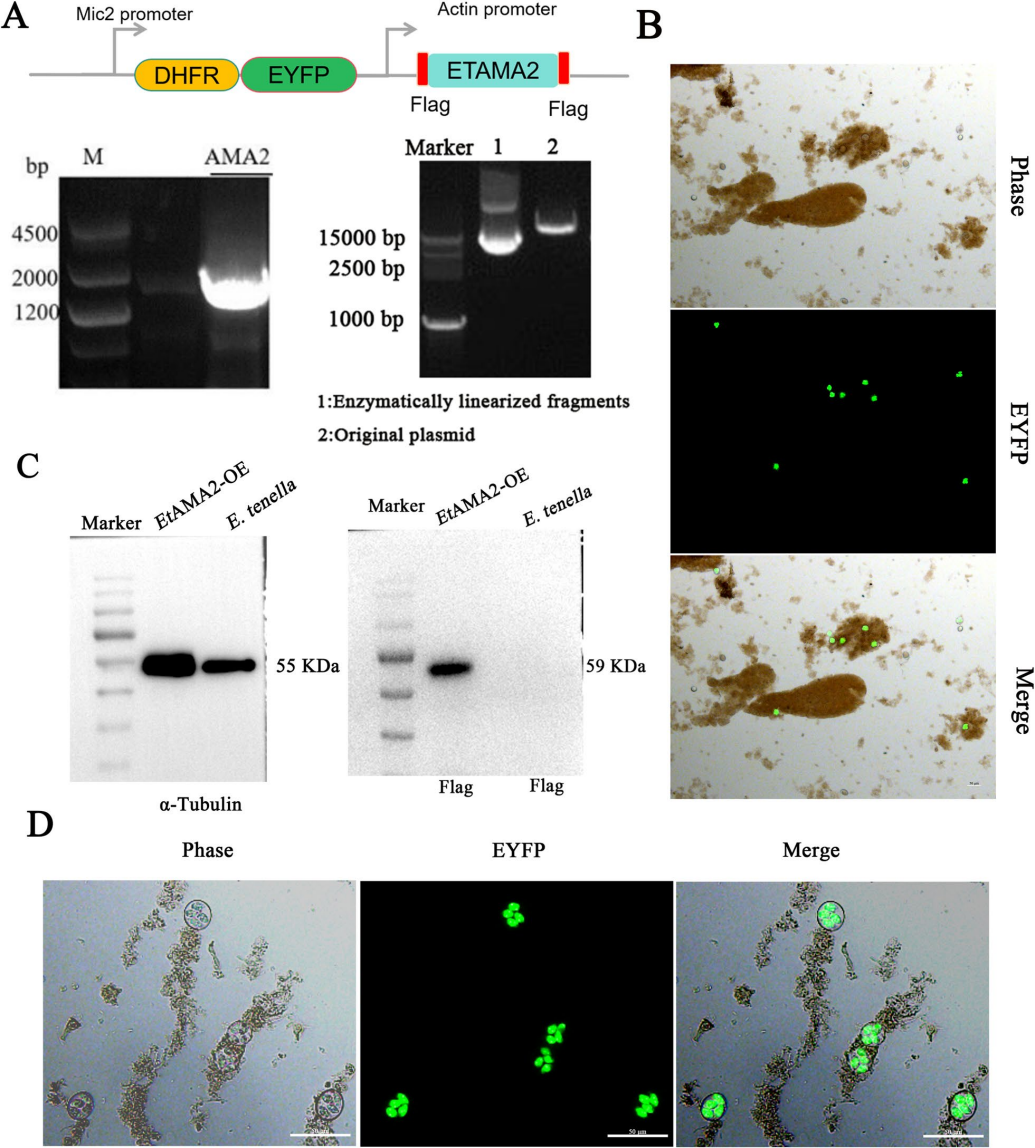
Construction and identification of the *Et*AMA2-OE strain. **A** Construction of the *Et*AMA2-OE plasmid. The left DNA electrophoresis figure shows the size of the gene encoding region of *Et*AMA2, while the right one represents the linear fragment after enzymatic digestion of the *Et*AMA2-OE plasmid. Schematic diagram of the *Et*AMA2 gene-overexpression vector. The vector contains two expression cassettes: the *E*tMic2 promoter drives the expression of mCherry fluorescent protein fused to DHFR, and the *Et*Actin promoter drives the expression of the target gene tagged with N- and C-terminal Flag peptides (shown in red). **B** Fluorescence microscopy observe EYFP^+^ green fluorescence from chicken cecum infected with transfected sporozoites (scale bars = 50 μm). **C** Western blotting by anti-Flag antibody showing *Et*AMA2 expression. **D** The homozygous *Et*AMA2-OE strain was obtained by single oocyst isolation technique (scale bars = 50 μm)

To investigate the role of *Et*AMA2 in *E. tenella* infection of host cells, we amplified the target gene *Et*AMA2 using purified cDNA from *E. tenella* as a template and successfully constructed the *Et*AMA2-OE plasmid (Fig. 2A). This plasmid was then introduced into *E. tenella* sporozoites and inoculated into chicks, with cecal oocysts collected at 7 days postinfection. The oocysts containing EYFP^+^ green fluorescence were observed using fluorescence microscopy (Fig. 2B). To obtain homozygous *Et*AMA2-OE strains, we used 300 ppm of pyrimethamine for drug selection and sorted EYFP^+^ green fluorescent oocysts by flow cytometry. Finally, the chicks were infected with an oocyst using the single oocyst isolation technique to obtain the homozygous *Et*AMA2-OE strain. Fluorescence microscopy revealed that all four sporocysts within each oocyst exhibited complete YFP-positive fluorescence, and Western blot analysis confirmed the successful overexpression of *Et*AMA2 in *E. tenella*. These results collectively demonstrate the successful generation of a homozygous *Et*AMA2-OE strain. (Fig. 2C, D).

### Pathogenicity of the *Et*AMA2-OE strain significantly enhanced

**Fig. 3:**
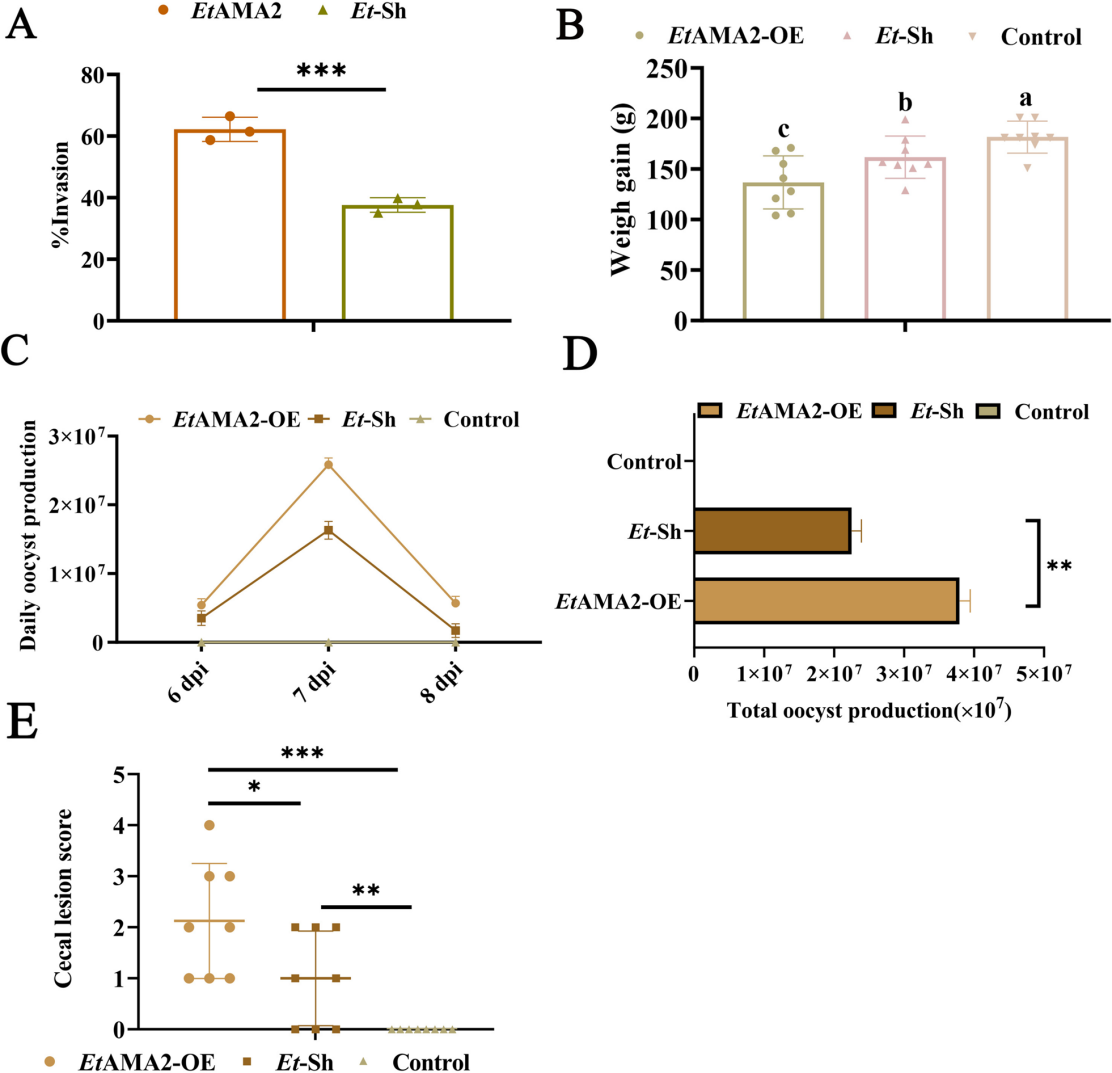
The pathogenicity of *Et*AMA2-OE strain was enhanced. **A** The effects of *Et*AMA2-OE strain on the invasion of DF-1 cells. DF-1 cells were inoculated into 12-well culture plates and then exposed to equal numbers of *Et*AMA2-OE or *Et*-sh strains sporozoites for 8 h(cell:sporozoite = 1:3). Sporozoite invasion rates were detected with flow cytometry (*n* = 3 biologically independent samples). **B** The effect of infection with *Et*AMA2-OE on the body weight of chicks. The weights of the chicks were measured (WG = final BW − initial BW). **C** and **D** Oocyst production in chicks infected with the *Et*AMA2-OE strain. Apart from the healthy control group, the other two groups were orally inoculated with equal amounts of sporulated oocysts of either the *Et*-Sh or *Et*AMA2-OE strain (2.0 × 104 oocysts per chick). Feces were collected, and oocyst production was assessed (oocysts per gram of stool [OPG]): total oocyst production = total of daily oocyst shedding per bird from 6 to 8 dpi; daily oocyst shedding per bird = OPG × fecal weight/number of birds. **E** The effect of infection with *Et*AMA2-OE on the chicks’ cecal lesions. Cecal lesions were scored according to the method of Johnson and Reid (1970); chickens that died from coccidiosis during the experiment were assigned a cecal lesion score of 4. All data presented are the means ± SD of three independent experiments. Diferent groups were compared with a t test ( *p<0.05,**p<0.01,***p<0.001,^a,b,c,d^*p*<0.05)

To investigate the impact of the *Et*AMA2 on the parasite’s pathogenicity, we examined the invasion rate of DF-1 cells by independently infecting them with the same number of sporozoites from *Et*AMA2-OE and *Et*-sh strains labeled with Vybrant^®^ CFDA-SE fluorescent dye. A flow cytometry analysis revealed that the invasion rate of the *Et*AMA2-OE strain was significantly higher than that of the *Et*-sh strain (Fig. 3A). Further assessments were conducted on cecal lesions, oocyst production, and body weights of chicks infected with the *Et*AMA2-OE strain. Compared with the healthy control group, both the *Et*-sh and *Et*AMA2-OE groups experienced significant weight loss, with the latter displaying a markedly greater weight reduction than the former (*P* < 0.05; Fig. 3B; Supplementary Table S1). Additionally, oocyst shedding in the *Et*AMA2-OE infection group was significantly higher than in the *Et*-sh infection group at 7 days postinfection, with total oocyst output from the *Et*AMA2-OE group substantially exceeding that of the *Et*-sh group (*P* < 0.01; Fig. 3C, D; Supplementary Table S2). The cecal lesion score was notably higher in the *Et*AMA2-OE infection group than in the *Et*-sh infection group (*P* < 0.01), indicating that *Et*AMA2-OE infection exacerbated the severity of cecal lesions in chicks (Fig. 3E; Supplementary Table S3). These experimental results indicated that the pathogenicity of the *Et*AMA2-OE strain was significantly enhanced compared with that of the *Et*-sh strain.

### The *Et*AMA2-OE strain conferred measurable immune protection to chicks against challenge

**Fig. 4:**
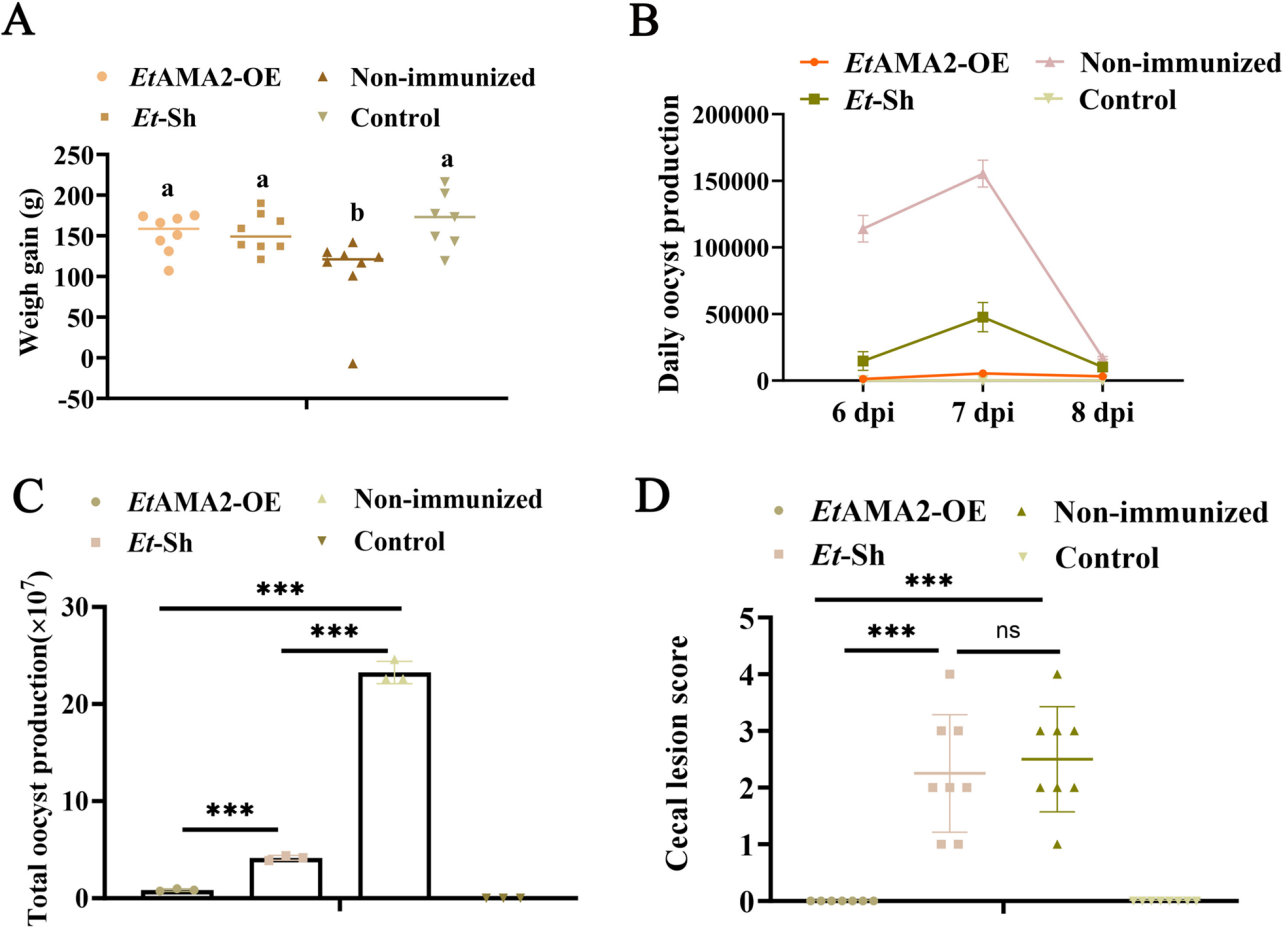
*Et*AMA2-OE strain provided strong immune protection to chicks. The 7-day-old chicks were inoculated with 1 × 10^3^ oocysts of either the *Et*AMA2-OE or *Et*-sh strains and subsequently challenged them with 2 × 10^4^ oocysts of *Et*-Sh at 14 days post-immunization. The changes in body weight, oocyst excretion, and cecal lesions were evaluated. **A** Weight changes of chicks after *Et*-Sh challenge (WG = final BW – initial BW). Daily OPG **B** and total oocyst production **C** of chicks after *Et*-Sh challenge. Total oocyst production = total of daily oocyst shedding per bird from 6 to 8 dpi; daily oocyst shedding per bird = OPG × fecal weight/number of bird; total oocyst production = total of daily oocyst shedding per bird from 6 to 8 dpi; daily oocyst shedding per bird = OPG × fecal weight/number of bird (*n* = 3 three oocyst count results). **D** Cecal lesion score of chicks after *Et*-Sh challenge. All data presented are the means ± SD of three independent experiments. Diferent groups were compared with a t test ( *p<0.05,**p<0.01,***p<0.001,^a,b,c,d^ *p*<0.05).

To assess the immunogenicity of the *Et*AMA2-OE strain, we inoculated 7-day-old chicks with 1 × 10^3^ oocysts of either the *Et*AMA2-OE or *Et*-sh strains and subsequently challenged them with 2 × 10^4^ sporulated oocysts of *Et*-Sh at 14 days post-immunization. The changes of body weight, oocyst excretion and cecal lesions were evaluated. Compared with the non-immunized infection group, the body weights of chicks in the immunized group increased significantly (*P* < 0.05); however, there was no significant change in weight gain between the *Et*AMA2-OE immunized and *Et*-Sh immunized groups (*P* > 0.05) (Fig. 4A; Supplementary Table S4). Both daily and total oocyst excretions in the *Et*AMA2-OE immunized group were significantly lower than in the other group (*P* < 0.001) (Fig. 4B, C; Supplementary Table S5). Additionally, the *Et*AMA2-OE immunized group exhibited almost no cecal lesions, resembling the healthy control group (*P* > 0.05), whereas the other group showed significantly higher numbers of cecal lesions (*P* < 0.001; Fig. 4D; Supplementary Table S6). These results indicated that the EtAMA2-OE strain possesses enhanced immunogenicity and confers robust protection against *E. tenella* challenge in chickens.

## Discussion

The AMA family in apicomplexa forms a group of highly conserved microneme proteins that are primarily involved in parasite invasion, pathogenesis, immune evasion, and other key functions [20, 30, 32]. The expression patterns of *Et*AMA family homologs vary across different *E. tenella* developmental stages, indicating that they perform distinct functions [30, 33, 34]. In this study, we used RT-qPCR to measure the mRNA transcriptional levels of *Et*AMA2 at various developmental stages of *E. tenella* infection in chicken ceca. *Et*AMA2 exhibited a high mRNA transcriptional level during the early infection phase, peaking at 24 hpi, which suggested that its functional role was confined to the invasion and schizogony stages, with minimal impact on subsequent developmental processes. Apicomplexan protozoan AMA proteins are commonly associated with host cell invasion and adhesion processes in parasites [19, 35]. For example, AMA1 proteins are involved in host cell recognition and invasion processes in *Plasmodium* and *T. gondii* [18, 36, 37]. Similarly, *Et*AMA2 may perform these, or related, functions when *E. tenella*’s sporozoites invade a host. These findings suggested that the differential high expression of *Et*AMA2 may be related to specific stages in the life cycle of *E. tenella*. To investigate this hypothesis, we successfully constructed an *Et*AMA2-OE strain by randomly inserting linearized DNA fragments into the *E. tenella* genome, resulting in high expression of the *Et*AMA2 [38, 39]. Functional characterization confirmed that *Et*AMA2-OE significantly enhanced sporozoite invasion, suggesting that *Et*AMA2 played a pivotal role in the invasion process. This observation aligned with findings in *T. gondii*, in which the expression, or absence, of specific invasion-associated proteins has been demonstrated to modulate invasion efficiency [18, 23].

A higher invasion rate likely facilitates the successful infection of a greater number of host cells by the parasite, leading to exacerbated tissue damage and pathological manifestations [40]. In this study, the *Et*AMA2-OE strain showed a significant increase in cell invasion capability and caused notable intestinal damage in chicks. This indicated that the enhanced virulence and pathogenicity observed for the *Et*AMA2-OE strain may be attributed to the improved invasion efficiency. Furthermore, *Et*AMA2 may contribute to increased virulence by modulating host cell immune responses. For example, AMA1 in *Plasmodium* is known to regulate host cell signaling pathways, thereby suppressing immune responses and promoting parasite survival [41]. This suggested that *Et*AMA2 could augment the virulence of *E. tenella* through a comparable mechanism, such as the disruption of host immune signaling pathways or the evasion of immune detection. Collectively, our findings indicate that *Et*AMA2 not only facilitates host cell invasion but also functions as a virulence factor.

Due to the strong antigenicity and immunogenicity of AMAs in apicomplexa, they have been proposed as potential vaccine targets against these parasites [27, 41]. For instance, *Babesia bovis* AMA-1 contains conserved B- and T-cell epitopes that generate neutralizing antibodies and a long-lasting Th1 immune response in vaccinated cattle [42]. Additionally, a parasite-specific Th1 immune response has been successfully induced in pregnant mice immunized with M3-*Neospora caninum* AMA1, effectively reducing offspring mortality from *N. caninum* infection [43]. A recombinant vaccine of *Et*AMA1 protein antigen expressed by *Lactococcus* has been constructed and demonstrated potency in enhancing the anticoccidial efficiency from a medium to a high level [27]. This suggested that the rational use of an antigen delivery vector was of great significance for exploring the immunodominant antigens of *Eimeria*. Transgenic *Eimeria* strains, constructed through either stable or transient transfection, are currently being explored as novel delivery vehicles for the immunodominant antigens of *Eimeria* [44]. In this study, we found that chicks immunized with the *Et*AMA2-OE strain and subsequently re-infected with wild strains exhibited significantly reduced daily and total oocyst counts, with cecal lesions that were very mild or even indistinguishable from those in healthy chicks, this was in stark contrast to the severe lesions (score 2.43 ± 0.97) observed in the non-immunized control group. On the basis of the prediction of multiple B- and T-cell epitopes in the *Et*AMA2 protein, we hypothesize that it may exert immunoprotective effects by activating both humoral and cellular immune responses, employing mechanisms similar to those described for the AMA1 protein of *N. caninum* [42]. To further elucidate the mechanism of immune protection induced by the *Et*AMA2-OE strain, subsequent studies will implement detailed immunophenotyping of the cecal mucosal immune response. In addition, it is noteworthy that the average cecal lesion score of the wild-type *E. tenella*-infected group in the initial pathogenicity test was 1.00 ± 0.86, which is lower than that of the non-immunized control group in the immune protection trial. This variation in baseline pathology between the two control groups is a common occurrence in animal studies and can be attributed to factors such as variability between different batches of chicks, oocyst storage time, and environmental factors. Crucially, despite this variation, the relative protection afforded by the *Et*AMA2-OE vaccine was consistently significant when compared with the respective control group within each experiment. These results indicate that immunization with the *Et*AMA2-OE strain provided consistent protection under the experimental conditions.

We hypothesized that the increased immunogenicity of the *Et*AMA2-OE strain was related to the high expression and exposure during invasion. Furthermore, the increased invasion efficiency of the *Et*AMA2-OE strain may result in more parasite antigens being presented to the host’s immune system. For example, chickens vaccinated with double-transgenic *Eimeria* parasites, expressing both AMA1 and IMP1 antigens, exhibit reduced oocyst output and enhanced cellular immune responses, indicating that higher protection levels were observed when both antigens were delivered [45]. These findings suggest that the elevated invasion efficiency of the *Et*AMA2-OE strain delivers more antigens to stimulate the immune response progression. Although the present study did not directly evaluate the immunogenic potential of purified recombinant *Et*AMA2 (r*Et*AMA2) in chickens, a previously published report demonstrated that r*Et*AMA2 expressed in *E. coli* failed to confer protective immunity against *E. tenella* challenge [46]. The apparent discrepancy between our results and those of the earlier study may be explained by several critical factors. First, recombinant proteins produced in prokaryotic expression systems such as *E. coli* frequently lack eukaryotic post-translational modifications (e.g., glycosylation) and often exhibit improper folding, thereby compromising their antigenic integrity. This is particularly relevant given that studies of AMA1 in *Plasmodium* parasites have established that its immunogenic activity is strictly dependent on correct conformational structuring [47]. Second, protective immunity against coccidiosis requires robust cell-mediated responses—critical for controlling intracellular stages—as well as mucosal immunity within the gut. Thus, future studies should employ eukaryotic expression systems to produce properly modified and folded *Et*AMA2, combined with optimized antigen delivery strategies and immunization protocols, to comprehensively evaluate the vaccine potential of *Et*AMA2.

## Conclusions

The overexpression of *Et*AMA2 significantly increased the parasite’s invasion efficiency and virulence, while also boosting its immunogenicity. These finding suggest that *Et*AMA2 may represent a potential candidate for further evaluation as a vaccine antigen. Future studies should focus on elucidating the molecular mechanisms of its interaction with host cells and its precise role in eliciting protective immune responses.

## Supplementary Information

Supplementary Material 1.

## Data Availability

Data supporting the main conclusions of this study are included in the manuscript.
