# Supplementary material for: Eimeria tenella apical membrane 2 overexpression enhances pathogenicity and immunity in chickens
Source: Parasit Vectors. 2025 Dec 6;19:20. doi: 10.1186/s13071-025-07185-0 (PMC12797683; doi:10.1186/s13071-025-07185-0)

**Supplementary information**

**Table 1:** Body weight gain of chicks infected with the *Et*AMA2-OE or *Et*-Sh strain

| Groups  (n=8) | Infection dose | Initial BW  (g) | Final BW  (g) | BWG  （g） | RBWG（%） |
| --- | --- | --- | --- | --- | --- |
| *Et*AMA2-OE | 2×10^4^ | 224±19 | 361±27 | 136±16**^c^** | 75 |
| *Et*-Sh | 2×10^4^ | 222±16 | 384±23 | 161±20**^b^** | 89 |
| Control | -- | 223±20 | 405±26 | 181±15**^a^** | 100 |

BWG: Weigh gain = Final average BW- Initial average BW

RWG: Relative weight gain rate=( average weight gain of the *Et*AMA2-OE or *Et*-Sh group / average weight gain of the control group)×100%

**Table 2:** Oocyst production of chicks infected with the *Et*AMA2-OE or *Et*-Sh strain

| Groups  (n=8) | Infection dose | 6 dpi  ×10^7^ | 7 dpi  ×10^7^ | 8 dpi  ×10^7^ | Total oocyst  ×10^7^ | Relative oocyst production rate |
| --- | --- | --- | --- | --- | --- | --- |
| *Et*AMA2-OE | 2×10^4^ | 0.54±0.09 | 2.68±0.11 | 0.57±0.09 | 3.79±0.12 | 165% |
| *Et*-Sh | 2×10^4^ | 0.35±0.10 | 1.7±0.10 | 0.17±0.09 | 2.29±0.16 | 100% |
| Control | -- | 0 | 0 | 0 | 0 | 0 |

The number of oocyst production by each chicken per day = (OPG × fecal weight) /Chicken number

Total oocyst: the total number of oocyst production by each chicken per day

Relative oocyst production rate = (average oocyst production of the *Et*AMA2-OE group/average oocyst production of the *Et*-Sh group)×100%

**Table 3:** Cecal lesions of chicks infected with the *Et*AMA2-OE or *Et*-Sh strain

| Groups  (n=8) | Cecal lesion  score for each bird | Average cecal lesion score |
| --- | --- | --- |
| *Et*AMA2-OE | 1、3、2、1、4、3、2、1 | (1.87±1.12)^c^ |
| *Et*-Sh | 2、2、2、1、0、1、0、0 | (1.00±0.86)^b^ |
| Control | 0、0、0、0、0、0、0、1 | (0.00±0.00)^a^ |

**Table 4:** Weight changes of chicks after immunization and challenge with *Eimeria tenella*

| Groups  (n=8) | Immunized strain | Immunized dose | Challenged strain | Challenged  dose | BWG  （g） | RBWG  （%） | |
| --- | --- | --- | --- | --- | --- | --- | --- |
| *Et*AMA2-OE | *Et*AMA2-OE | 1×10^3^ | *Et*-Sh | 2×10^4^ | （152.37±24.20）^a^ | | 90 |
| *Et*-Sh | *Et*-Sh | 1×10^3^ | *Et*-Sh | 2×10^4^ | （153.50±23.71）^a^ | 91 | |
| Non-immunized | -- | -- | *Et*-Sh | 2×10^4^ | （106.37±47.28）^b^ | 63 | |
| Control | -- | -- | -- | -- | （168.42±34.03）^a^ | -- | |

BWG = Average body weight at the time of slaughter - Average body weight at the time of challenge

**Table 5:** Oocyst excretion of chicks after immunization and challenge with *Eimeria tenella*

| Groups  (n=8) | Immunized  strain | Immunized dose | Challenged strain | Challenged dose | Total oocyst | Relative oocyst production rate |
| --- | --- | --- | --- | --- | --- | --- |
| *Et*AMA2-OE | *Et*AMA2-OE | *Et*-Sh | 1×10^3^ | 2×10^4^ | (0.83±0.13)×10^7^ | 4% |
| *Et*-Sh | *Et*-Sh | *Et*-Sh | 1×10^3^ | 2×10^4^ | (4.19±0.25)×10^7^ | 19% |
| Non-immunized | -- | *Et*-Sh | -- | 2×10^4^ | (22.6±1.65)×10^7^ | 100% |
| Control | -- | -- | -- | -- | -- | -- |

Relative oocyst production rate = (average oocyst production of the experimental group/average oocyst production of the infection non-immunized group)×100%

**Table 6:** Cecal lesion score of chicks after after immunization and challenge with *Eimeria tenella*

| Groups  (n=8) | Immunized  strain | Immunized dose | Challenged strain | Challenged dose | Cecal lesion  score for each bird | Average cecal lesion score |
| --- | --- | --- | --- | --- | --- | --- |
| *Et*AMA2-OE | *Et*AMA2-OE | 1×10^3^ | *Et*-Sh | 2×10^4^ | 0、0、0、0、0、0、0、0 | （0.00±0.00） |
| *Et*-Sh | *Et*-Sh | 1×10^3^ | *Et*-Sh | 2×10^4^ | 2、2、3、4、3、1、1、2 | （2.25±1.03） |
| Non-immunized | -- | -- | *Et*-Sh | 2×10^4^ | 4、3、2、1、2、2、3、3 | （2.43±0.97） |
| Control | -- | -- | -- | -- | 0、0、0、0、0、0、0、0 | （0.00±0.00） |

**Table 7.** Primer sequences used in qPCR

| **Target** | **sense** |
| --- | --- |
| *Et*AMA2-F | GCCGCTCTTATCGCCTACTTCATC |
| *Et*AMA2-R | CTCCTCATCCCTCTCCTCGTCTTC |
| *Et18S rRNA* -F | TGTAGTGGAGTCTTGGTGATTC |
| *Et18S rRNA* -R | CCTGCTGCCTTCCTTAGATG |

**Table 8.** Primer sequences used for constructing the *Et*AMA2-OE vector

| **Target** | **sense** |
| --- | --- |
| *Et*AMA2-F | ATGGAGGCTCTACGGGAAGGCTTC |
| *Et*AMA2-R | GTAGTAGGCGTCGTGGGCGTT |
| *Et*AMA2-OE-F | TCTAGAATGGATTACAAGGATGACGACGATAAGATGGAGGCTCTACGGGAAGGCTTC |
| *Et*AMA2-OE-R | CCGCGGTCACTTATCGTCGTCATCCTTGTAATCGTAGTAGGCGTCGTGGGCGTTGTG |

**Supplementary Fig. 1 *Et*AMA2-OE plasmid profile**


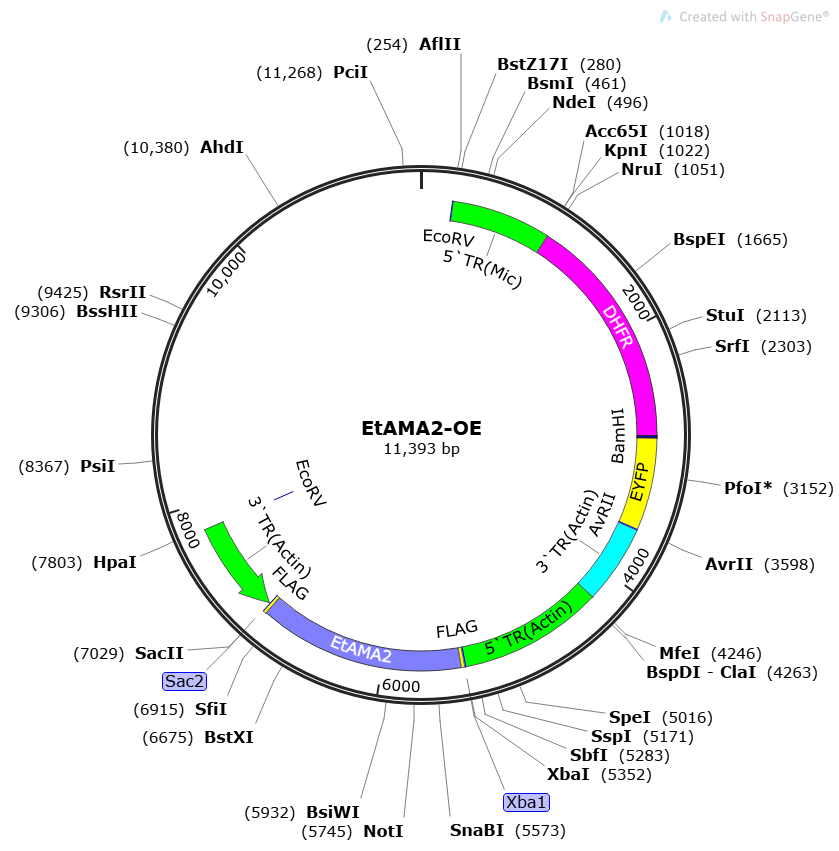

Supplement: Supplementary file 1 — Supplementary Material 1. [file 13071_2025_7185_MOESM1_ESM.docx]
